# Multi illumination color constancy based on multi-scale supervision and single-scale estimation cascade convolution neural network

**DOI:** 10.3389/fninf.2022.953235

**Published:** 2022-12-09

**Authors:** Fei Wang, Wei Wang, Dan Wu, Guowang Gao, Zetian Wang

**Affiliations:** ^1^School of Electronic Engineering, Xi'an Shiyou University, Xi'an, China; ^2^State Key Laboratory of Advanced Design and Manufacturing for Vehicle Body, Hunan University, Hunan, China; ^3^School of Telecommunications Engineering, Xidian University, Xi'an, China

**Keywords:** color constancy, multi-illumination, convolution neural network, cascade, multi-scale

## Abstract

Color constancy methods are generally based on a simplifying assumption that the spectral distribution of a light source is uniform across scenes. However, in reality, this assumption is often violated because of the presence of multiple light sources, that is, more than two illuminations. In this paper, we propose a unique cascade network of deep multi-scale supervision and single-scale estimation (CN-DMS4) to estimate multi-illumination. The network parameters are supervised and learned from coarse to fine in the training process and estimate only the final thinnest level illumination map in the illumination estimation process. Furthermore, to reduce the influence of the color channel on the Euclidean distance or the pixel-level angle error, a new loss function with a channel penalty term is designed to optimize the network parameters. Extensive experiments are conducted on single and multi-illumination benchmark datasets. In comparison with previous multi-illumination estimation methods, our proposed method displays a partial improvement in terms of quantitative data and visual effect, which provides the future research direction in end-to-end multi-illumination estimation.

## 1. Introduction

With the rapid proliferation of digital imaging and digital video, accurate recording of the constant color of a scene from the device-captured image is of extreme importance for many practical applications, ranging from color-based object recognition and tracking to quality control of textiles (Funt et al., 1999; Vrhel et al., 2005; Gao et al., 2017, 2019). The color of an object is influenced by the illumination color and the observed color of an object in an image (representing the observed values in RGB space) depends on the intrinsic color and light-source color (Ebner, 2007).

Human color constancy (HCC) is a perceptual phenomenon that stabilizes the appearance of an object's colors throughout changes in illumination. One possible-ecological justification for color constancy in mammals is to facilitate scene object recognition (Kraft and Brainard, 1999; Smithson, 2005). In Helmolt's words: “Colors are mainly important for us as properties of objects and as means of identifying objects.” Then a mechanism that preserves the color appearance of objects will serve this purpose. As a perceptual phenomenon, all variables affecting color constancy lie in the content of the perceived scene, e.g., scene chromaticity, three dimensional information, object movement, and some others. All these factors are called visual cues (Jameson, 1989; Roca-Vila et al., 2009). Numerous tests of human perception of colored surfaces indicate a high level of perceptual constancy, in which the appearance of the surface is relatively little changed. However, endowing a computer with the same ability is difficult (Gilchrist, 2006; Roca-Vila et al., 2009). To assist a computer in solving this problem, our central problem is to estimate the real object's color coordinates in some color space, which is called computational color constancy (CCC).

Previous methods have mostly been limited to a single-illumination assumption. However, in reality, most scenes have more than one illumination. In multi-illumination scenes, each pixel in an image is influenced by different light sources, unlike that of single illumination. For example, in an image with shadows, there are at least two lights (the light colors of different degrees of shadow areas and normal sunlight areas are different). Therefore, research on multi-illumination color constancy (MCC) has more practical significance.

However, fewer studies have been conducted on MCC than on single illumination. This is mainly because it is difficult to obtain datasets for multiple lighting conditions, especially for lighting colors requiring manual calibration of pixel-level accuracy.

As with single illumination, MCC methods can be classified into optimization- and learning-based methods.

**Optimization-based methods:** Land et al. first proposed the Retinex model (Brainard and Wandell, 1986; Land, 1986; Funt et al., 2004), which is the earliest theoretical model that can deal with the MCC problem. This theory is based on a series of psychological and physical experiments. The early purpose was not to estimate the illumination under multiple illumination conditions but to restore the relative reflectivity of objects in a scene. Barnard et al. (1997) proposed a model to deal with the MCC problem by detecting the change of illumination color in the scene. The model is patch-based and estimates the illumination of an image patch through single-illumination color constancy. Xiong and Funt (2006) used a diffusion technique in which a large-scale convolution kernel is used to filter the color-biased images in complex scenes. It is assumed that the images after convolution meet the local gray-world assumption. Although this method has achieved good results, it only uses simple convolution kernels that are easily affected by the real color of the object itself. For example, part of the obtained illumination map is the color of the object itself, rather than the illumination color.

**Learning-based methods:** Like other data mining tasks, this method learns useful information from large amounts of data (Barnard et al., 2010; Kannimuthu et al., 2012; Arunkumar et al., 2019). Shi et al. (2016) and Bianco et al. (2017) used patch-based convolutional neural networks (CNNs) to estimate a single illumination for each patch. By inputting each patch into the network, the local illumination of all patches can be obtained. Afifi and Brown (2020) proposed an end-to-end approach to learning the correct white balance, which consists of a single encoder and multiple decoders, mapping an input image into two additional white-balance settings corresponding to indoor and outdoor illuminations. This method can also be used in multi-illumination estimation; however, our experiments show that it is very time-consuming.

The abovementioned multi-illumination and single-illumination estimation methods have achieved good performance on some multi-illumination datasets. However, these methods may not find the optimal solution in some complex situations owing to their inflexibility. To summarize, there are still some unsolved open problems in these approaches, which can be generally summarized as two aspects:

- Many of these methods (Xiong and Funt, 2006; Zeng et al., 2011; Mutimbu and Robles-Kelly, 2016) are implemented by clustering the illumination of local regions. However, the process of clustering is a difficult problem. If the illumination distribution in the scene is scattered, then it is difficult to obtain accurate illumination. In addition, the selection of region size is also a key problem. Inappropriate region size will reduce the accuracy of illumination estimation, and these methods are based on the traditional assumption of illumination estimation. If the region does not meet this assumption, the corresponding regional illumination estimation may be in error.
- Most existing CNN-based single-illumination estimation methods used for multi-illumination estimation are time-consuming (Barron, 2015; Shi et al., 2016; Bianco et al., 2017) when adopting the local image patches for estimation.

In recent years, CNNs have been widely used, especially the fully convolutional networks for image pixel classification (Shelhamer et al., 2014; Yu and Koltun, 2015; Badrinarayanan et al., 2017) and image depth estimation (Eigen et al., 2014; Eigen and Fergus, 2015), to improve the estimation accuracy to a new level. In multi-illumination estimation, the pixel-level illumination is estimated from the original color-biased image, which is consistent with the image segmentation scene and depth estimation scene (Eigen et al., 2014; Shelhamer et al., 2014; Yu and Koltun, 2015; Badrinarayanan et al., 2017).

In this paper, we propose a cascade network of deep multiscale supervision and single-scale estimation to estimate multi-illumination (CN-DMS4)^1^. For training, the parameters are learned from coarse to fine and through different scales. In the test phase, only the illumination map of the thinnest level is estimated.

The CN-DMS4 network differs from existing methods, and provides two contributions:

- Multiscale supervision and single-scale estimation. The network is an end-to-end cascaded structure; the network parameters are supervised and learned from coarse to fine during the training process. Only the final thinnest level illumination map is estimated in the illumination estimation process.
- A new loss function with a channel penalty term is designed to optimize the network parameters, which can solve the influence of color channels in the Euclidean distance or pixel-level angle error.

The remainder of this paper is organized as follows. In Section 2, the structure of the proposed network and training strategy are presented. The experimental results are provided in Section 3. The conclusion is given in Section 4.

## 2. Multi-scale supervision and single-scale estimation in a cascade convolutional neural network

Following the widely accepted simplified diagonal model (Finlayson et al., 1994; Funt and Lewis, 2000), we also use this model in our study. For multi-illumination estimation, we modify the diagonal model as follows:


(1) $$\begin{array}{l} I_{c}\left(x,y\right)=E_{c}\left(x,y\right)\times R_{c}\left(x,y\right),c\in \left\{r,g,b\right\}, \end{array}$$

where the illumination in the scene is *E*_*c*_(*x, y*), (*x, y*) is the spatial position in an image, *I*_*c*_(*x, y*) represents the image under unknown illumination, *E*_*c*_(*x, y*) represents the illumination image, and *R*_*c*_(*x, y*) represents the image under standard illumination.

### 2.1. Problem formulation

As in the single-illumination estimate, we only know the image *I*_*c*_(*x, y*) under an unknown light source *E*_*c*_(*x, y*), which needs to be estimated. The goal of multi-illumination estimation is to estimate *E*_*c*_(*x, y*) from *I*_*c*_(*x, y*), and then compute it as *E*_*c*_(*x, y*) = *I*_*c*_(*x, y*)/*R*_*c*_(*x, y*). To address the problem of estimating *E*_*c*_(*x, y*) from *I*_*c*_(*x, y*), we formulate it as a regression. A new color-space model, *log*−*uv*, has been used in color constancy methods (Finlayson et al., 2004; Barron, 2015; Shi et al., 2016) in recent years, and has certain advantages^2^. The calculation method is as follows:


(2) $$\begin{array}{l} L_{u}=log\left(R/G\right),L_{v}=log\left(B/G\right), \end{array}$$

After estimating the light, it can be converted back to the *RGB* space through a very simple formula:


(3) $$\begin{array}{l} \begin{array}{c} R=exp\left(-L_{u}\right)/z,G=1/z,B=exp\left(-L_{v}\right)/z,\\ z=\sqrt{\exp{\left(-L_{u}\right)}^{2}+\exp{\left(-L_{v}\right)}^{2}+1}, \end{array} \end{array}$$

where (*L*_*u*_, *L*_*v*_) is the image in the *log*−*uv* color space, and (*R, G, B*) is the image in the *RGB* color space.

In this study, we first convert the RGB image *I*_*c*_(*x, y*) to log-uv image *I*_*uv*_(*x, y*) = (*I*_*u*_(*x, y*), *I*_*v*_(*x, y*)). Our goal is to find a mapping *f*_*theta*_, such that *f*_θ_(*I*_*uv*_) = *E*_*uv*_(*x, y*), where *E*_*uv*_(*x, y*) represents the illumination value at each (*x, y*) in the *log*−*uv* space; *E*_*uv*_(*x, y*) should be as close as possible to the real light at the position of (*x, y*). In this paper, we define *f*_*theta*_ as a CNN model that is optimized by the parameter θ.

Based on the semantic segmentation model, we define the network into the encoding and decoding parts. The encoding part performs the process of feature extraction, and the decoding part performs the process of remapping these features back to the image. We define the encoding process by Equation (4), and the decoding the process by Equation (5):


(4) $$\begin{array}{l} Enc=\psi_{1}\left(I_{uv},\theta_{1}\right), \end{array}$$

(5) $$\begin{array}{l} Dec=\psi_{2}\left(Enc,\theta_{2}\right), \end{array}$$

where ψ_1_ represents the network of the encoding process, θ_1_ indicates the parameters to be optimized in the encoding part, ψ_2_ represents the decoding process, and θ_2_ indicates the parameters to be optimized in the decoding part.

In addition, refer to the idea in the literature (Mutimbu and Robles-Kelly, 2016), which uses a factor graph defined across the scale space of the input image and estimated the multi-illumination at multiple scales from fine to coarse (i.e., the image becomes increasingly blur), the pixelwise illuminant can be viewed as the geometric mean of the illuminants across all scales. In this paper, we also try to use a multiscale network to improve the estimation accuracy. The difference is that our method supervises and learns the parameters from coarse to fine (i.e., the image becomes increasingly clear).

### 2.2. Network architecture

**Figure 1:**
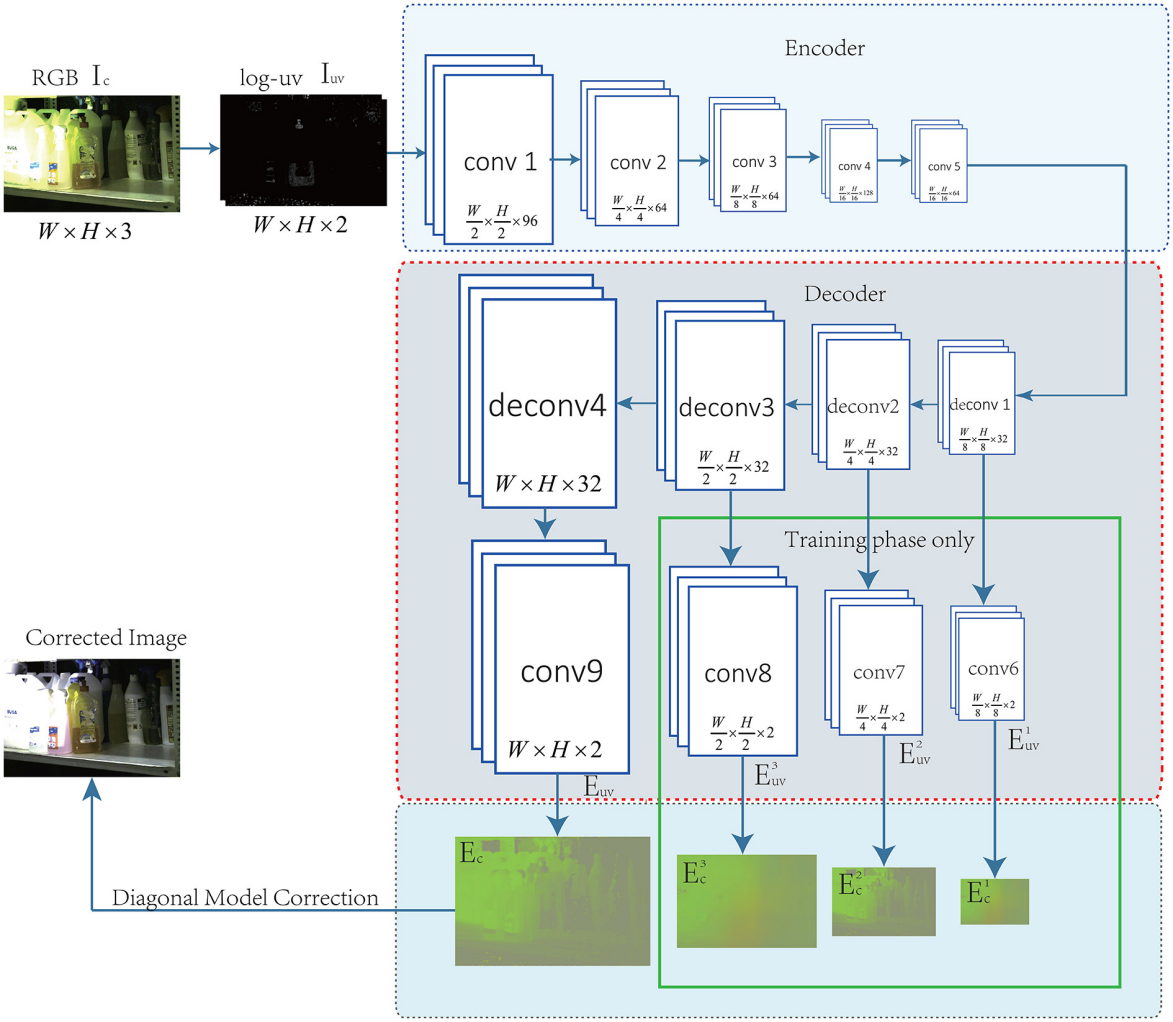
The network structure of CN-DMS4.

As introduced in the previous section, it is necessary to design a network structure that includes an encoding part ψ_1_ and decoding part ψ_2_. The network structure is shown in Figure 1.

**Encoding part of the network**. In Figure 1, the encoding part indicates ψ_1_ in Equation 4. The encoding part is used to extract features, which are then input to the decoding part to estimate the illumination. In this part, we also used AlexNet (Krizhevsky et al., 2017), VGGNet-16 (Simonyan and Zisserman, 2014), and VGGNet-19 (Simonyan and Zisserman, 2014), but the results showed little difference. Finally, we used the structure improved from AlexNet (Krizhevsky et al., 2017) containing 5 convolutions. We removed all the pooling layers and replaced them with a large stride of convolution kernels. All the layers use the convolution kernel of 3 × 3, and the stride of all the convolutions is set to 2.

**Decoding part of the network**. In Figure 1, the decoding part indicates ψ_2_ in Equation 5. The decoding part is used to reconstruct the pixel-level illumination. However, conv6, conv7, conv8, and conv9 use the convolution kernel of 1 × 1 to reduce the dimension, while the others use the convolution kernel of 3 × 3 and the stride is set to 2. In the training phase, in addition to *E*_*c*_, the ${E_{c}}^{1},{E_{c}}^{3},and\;{E_{c}}^{3}$ also participates in supervised learning. In the illumination estimation stage, illumination images at different scales can be obtained, or only the final and the finest illumination can be obtained but the part marked by the green box in Figure 1 cannot participate in the calculation.

### 2.3. Loss function

Our goal is to train a mapping function for generating an illumination image *E*(*u, v*) that is close to the ground-truth illumination image *E*_*t*_(*u, v*). Instead of minimizing the mean squared error between *E*(*u, v*) and *E*_*t*_(*u, v*) at each scale, we propose a variant of the L1. The overall loss function is defined as:


(6) $$\begin{array}{l} Loss=\frac{1}{N}\overset{N}{\underset{i=1}{\sum }}\overset{S}{\underset{j=1}{\sum }}\omega \left(\omega \left(E_{u}^{j}-E_{u\text{\_}t}^{j}\right)+\omega \left(E_{v}^{j}-E_{v\text{\_}t}^{j}\right)\right), \end{array}$$

where $\omega \left(x\right)=\sqrt{x^{2}+\varepsilon^{2}}$, *N* indicates the number of samples for each batch, *S* indicates the scale of the cascade, $E_{u}^{j}$, $E_{v}^{j}$ represents the illumination in the log-uv space estimated by the model at the *j* scale, $E_{u\text{\_}t}^{j}$ and $E_{v\text{\_}t}^{j}$ represents the ground truth at *j* scale, and ε takes the empirical value ε = 0.001.

## 3. Experimental results

### 3.1. Datasets

There are only a few public multi-illumination datasets, and the number of images in the datasets is limited. In the phase of network training, more data is needed. Based on the dissertation in Gao (2017), we use the single-illumination datasets Color Checker (Gehler et al., 2008) and NUS 8-Camera (Cheng et al., 2014) to render a large number of multi-illumination datasets.

**Figure 2:**
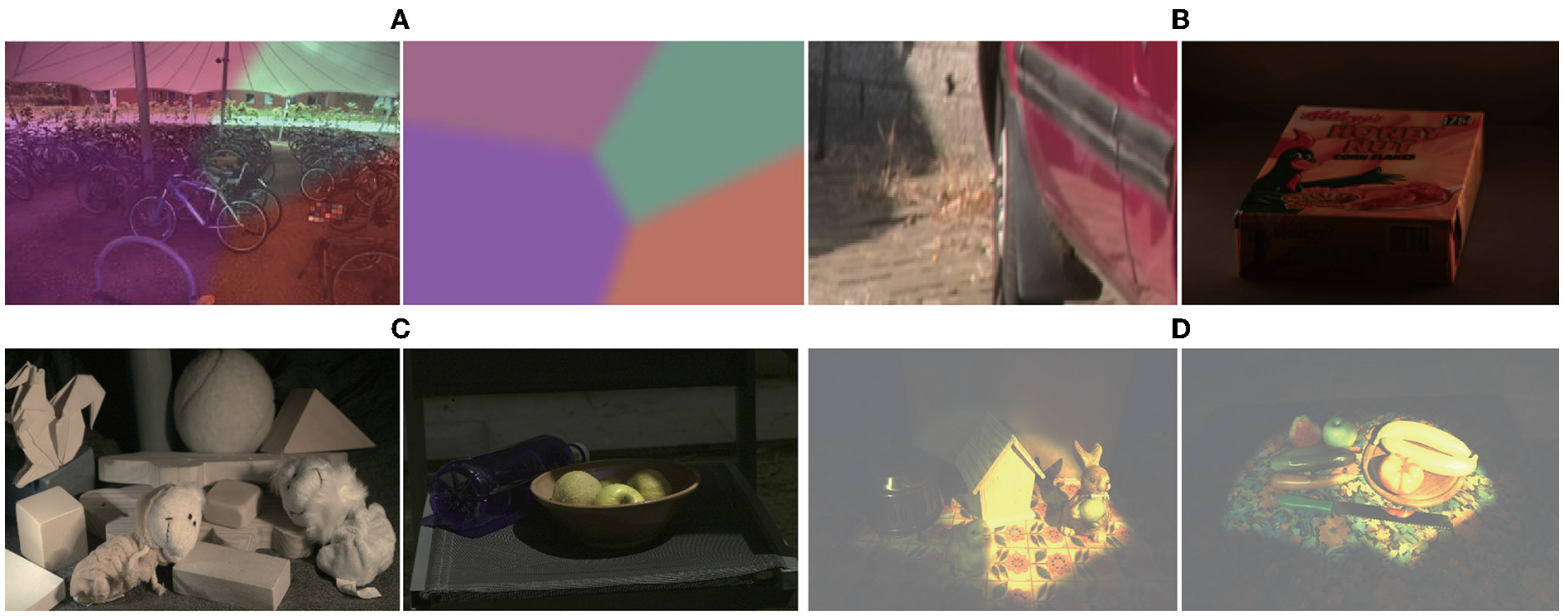
Images under multiple illuminations. From left to right: **(A)** Synthetic images; **(B)** Images from Gijsenij dataset: http://www.colorconstancy.com/wp-content/uploads/2014/10/multiple_light_sources_dataset.zip Reproduced with permission from Arjan et al. (2012); **(C)** Images from MIMO dataset available at: http://www5.cs.fau.de/research/data/two-illuminant-dataset-with-computed-ground-truth/. Reproduced with persmission from Beigpour et al. (2014); **(D)** Images from Bleier dataset available at: http://www5.cs.fau.de/research/data/multi-illuminant-dataset/index.html. Reproduced with permission from Bleier et al. (2011).

The operation process is as follows. First, the images are corrected to standard white light according to the illumination provided by the datasets. Next, multiple spatial positions are randomly generated on each image, and 3 − 8 different lighting colors are simulated, as shown in Figure 2A (the boundary is blurred).

In addition, the following multi-illumination datasets collected in real scenes are used, respectively. The Gijsenij dataset (Arjan et al., 2012), is a multi-illumination dataset collected in a natural scene that includes 59 indoor and 9 outdoor multi-illumination images, and their corresponding illuminations. Figure 2B shows an indoor and an outdoor image from this database.

The multiple-input multiple-output (MIMO) dataset (Beigpour et al., 2014) was established by Beigpour et al., which contains 57 indoor images and 21 outdoor images; it provides pixel-level illumination images. Figure 2C shows an indoor and an outdoor image from this database.

The Bleier dataset (Bleier et al., 2011) was collected and established by Bleier et al. The dataset contains 36 high-quality images and corresponding illumination images obtained by nine different illuminations in four scenes. Figure 2D shows two images from the database.

To enable the model to be used for single-light estimation, we added a single-light dataset, SFU Grayball dataset (Ciurea and Funt, 2003).

In addition, we utilize horizontal and vertical mirroring, rotating at [90^*o*^, 180^*o*^] and at [−60^*o*^, 60^*o*^] every five degrees, respectively. At the same time, we scale the data from [0.6, 1.5] times to obtain a total of 14,500 real scene datasets. We selected 5,000 images from the real multi-illumination dataset, 4,000 from the dataset that we constructed as training data, 3,000 from SFU Grayball dataset (Ciurea and Funt, 2003), and 2,500 from Shadow removal datasets (Zhu et al., 2010; Gong and Cosker, 2014; Sidorov, 2019). Finally, we resized these data to 512 × 512 as the input of the training network. Similar to most learning-based tasks, we used the 3-fold cross-validation.

### 3.2. Metrics

Similar to color constancy under single illumination, we use angular error to measure the performance of our MCC method. The difference is that we calculate the angular error pixel-by-pixel, and then average the angular error of the whole image. The angular error is defined by Equation (4).


(7) $$\begin{array}{l} err=\frac{1}{M\times N}\overset{N}{\underset{y=1}{\sum }}\overset{M}{\underset{x=1}{\sum }}\left(\arccos\left(\frac{E_{e}\left(x,y\right).E_{e}^{*}\left(x,y\right)}{\left||E_{e}\left(x,y\right)||.||E_{e}^{*}\left(x,y\right)|\right|}\right)\right) \end{array}$$

where *E*_*e*_(*x, y*) and $E_{e}^{*}\left(x,y\right)$ represents the estimated illumination and real illumination at position (*x, y*), respectively, and *M, N* represents the width and height of the image. The less the *err* is, the better the method performs.

Similar to previous multi-illumination estimate studies (Brainard and Wandell, 1986; Land, 1986; Barnard et al., 1997; Funt et al., 2004; Xiong and Funt, 2006; Zeng et al., 2011; Mutimbu and Robles-Kelly, 2016), we only compare the *mean* and *median* on multi-illumination datasets.

### 3.3. Implementation parameters

In this subsection, the parameter sets for training our final model are given.

**Encoding network selection:** Different network structures, such as AlexNet (Krizhevsky et al., 2017), VGGNet-16 (Simonyan and Zisserman, 2014), and VGGNet-19 (Simonyan and Zisserman, 2014), are used to test the performance. The network we designed (modified from AlexNet Krizhevsky et al., 2017) is slightly worse than VGGNet-19 (Simonyan and Zisserman, 2014), but the speed is more than 4 times faster than AlexNet (Krizhevsky et al., 2017) and VGGNet-19 (Simonyan and Zisserman, 2014). Finally, considering the effect and efficiency, the structure in Figure 1 is used in this study.

**Figure 3:**
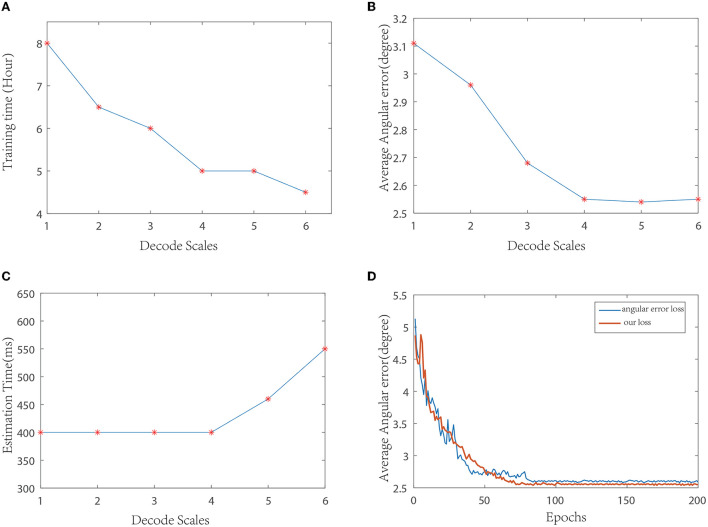
Performance curves under different parameters. **(A)** Comparison of training time of different decoding scales; **(B)** Comparison of average angular errors of different decoding scales; **(C)** Comparison of average time consumption of illumination estimation at different decoding scales; **(D)** Comparison of training curves of different loss functions.

**Decoding network selection:** The decoder part is equivalent to a process of feature reconstruction. The backbone network structure we used was symmetrical to the encoding network. We tested with different resized stages and compared the performance. The resulting curve is shown in Figures 3A– C. Finally, considering the effect and efficiency, the decoding structure shown in Figure 1 is used in this study. It can also be seen from the curve that under different resize levels of the decoder, the number of deconvolution layers does not increase, and the time consumptions of illumination estimations are essentially equal.

**Loss function selection:** During training, the angular error and loss function proposed in this study are compared, and the resulting curve is shown in Figure 3D. As can be seen from the curve, the loss function used in this paper converges faster than the angular error, and the training error is relatively smooth. At the same time, the test average error in several datasets is slightly lower than the angular error.

**Training parameters:** We used Adam (Kingma and Adam, 2014), and set *batch* = 64 to optimize the network in this work. The learning rate was set to 0.0001. Approximately 4, 000 epochs (total 906, 250 iterations at *batch* = 64) were performed.

### 3.4. Comparison with state-of-the-art methods

This paper is aimed at multi-illumination estimation. We compare the proposed method with some existing MCC methods and with some methods that can estimate local illumination, including the following three types.

One type consists of methods for which segmentation is not required, such as gray pixel (GP) (Yang et al., 2015), and a retinal neuron mechanism-based method proposed by Zhang et al. (2016).

The second type requires image segmentation, including the method of Arjan et al. (2012), the multi-illumination model proposed by Gu et al. (2014), and a multi-illumination estimation model based on the factor graph (FG) model (Mutimbu and Robles-Kelly, 2016).

The third type, developed in recent years, comprises single-illumination estimation methods based on CNNs, including the CNN method of Bianco et al. (2015) CC-CNN, DS-Net (Shi et al., 2016), and the grayness index (GI) (Qian et al., 2019).

**Figure 4:**
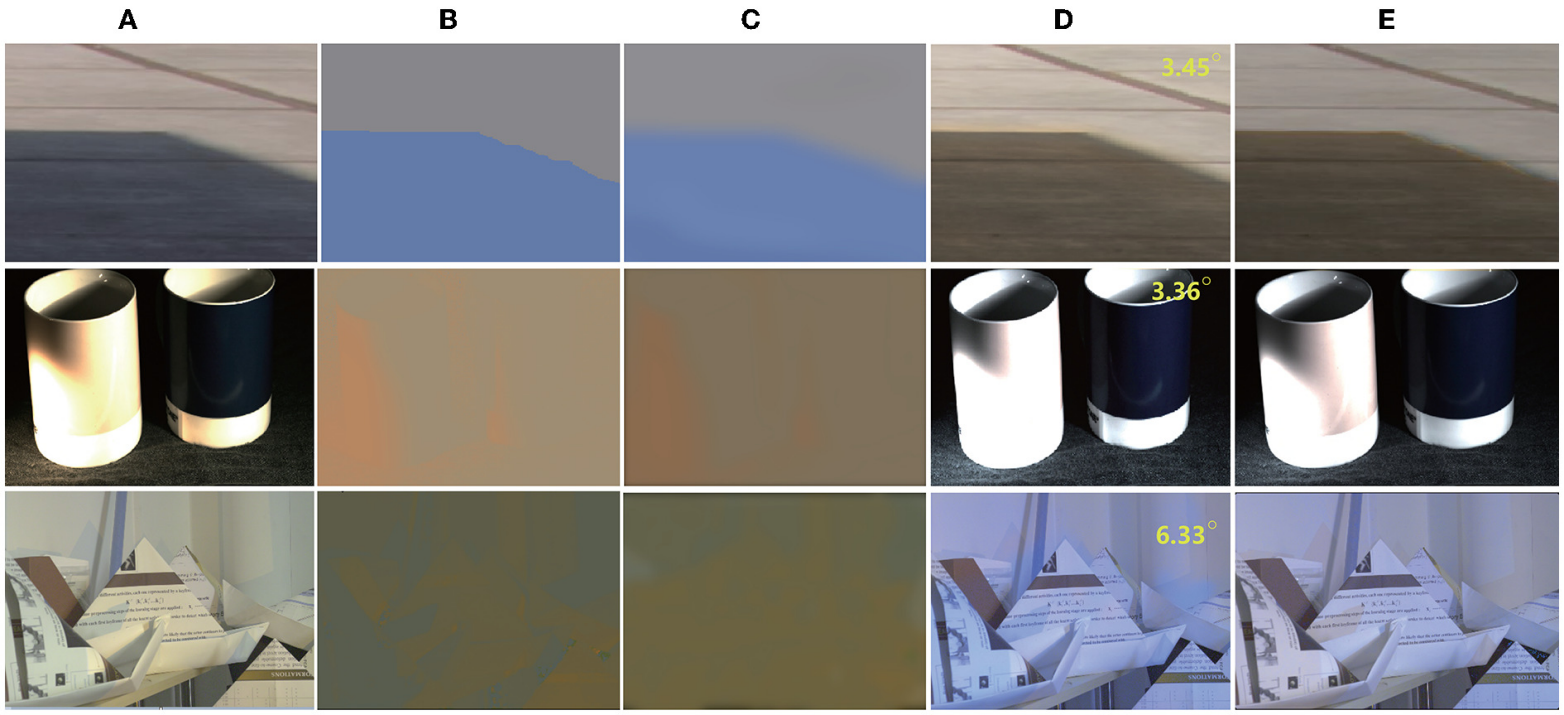
Qualitative results on MIMO multi-illumination datasets (Beigpour et al., 2014), the top right-hand corner of each image indicates the angle error. From left to right: **(A)** Original image; **(B)** Ground truth illumination image; **(C)** Estimated illumination image; **(D)** Corrected image; **(E)** Ground truth image. Dataset available at: http://www5.cs.fau.de/research/data/two-illuminant-dataset-with-computed-ground-truth/. Reproduced with permission from Beigpour et al. (2014).

The quantitative performance comparison on the Gijsenij dataset (Arjan et al., 2012) is presented in Table 1, the results on MIMO (Beigpour et al., 2014) in Table 2, and Bleier (Bleier et al., 2011) in Table 3. Some results are shown in Figure 4.

**Table 1:** Quantitative evaluation on the Gijsenij dataset (Arjan et al., 2012), red indicates the best.

	**Lab**		**Outdoor**	
**Method**	**Mean**	**Median**	**Mean**	**Median**
Retinex (Funt et al., 2004)	13.15	13.16	6.62	7.25
Zhang (Zhang et al., 2016)	14.64	14.48	8.45	8.23
GIJ-GW (Arjan et al., 2012)	11.7	-	6.4	-
GIJ-GE2 (Arjan et al., 2012)	12.4	-	5.1	-
GU-GE1 (Gu et al., 2014)	3.25	-	3.26	-
GU-WP (Gu et al., 2014)	2.97	-	3.20	-
FG (Mutimbu and Robles-Kelly, 2016)	2.68	-	3.10	-
CC-CNN (Bianco et al., 2015)	5.71	5.97	3.92	4.26
DS-Net (Shi et al., 2016)	3.76	4.13	4.60	4.80
CN-DMS4	2.51	2.58	2.39	2.41

**Table 2:** Quantitative Evaluation on MIMO (Beigpour et al., 2014), red indicates the best.

**MIMO dataset**	**Lab**		**Outdoor**	
**Method**	**Median**	**Mean**	**Median**	**Mean**
Retinex (Funt et al., 2004)	4.92	5.36	4.69	5.84
Zhang (Zhang et al., 2016)	2.71	3.21	4.35	5.18
GIJ-WP (Arjan et al., 2012)	4.2	5.1	3.8	4.2
GIJ-GE1 (Arjan et al., 2012)	4.2	4.8	9.2	9.1
GU-GE1 (Gu et al., 2014)	3.16	-	3.54	-
GU-GW (Gu et al., 2014)	3.86	-	4.43	-
FG (Mutimbu and Robles-Kelly, 2016)	2.96	-	3.48	-
CC-CNN (Bianco et al., 2015)	2.98	3.22	3.35	3.72
DS-Net (Shi et al., 2016)	3.21	3.46	3.01	3.86
CN-DMS4	2.50	2.83	2.99	3.33

**Table 3:** Quantitative evaluation on Bleier (Bleier et al., 2011), red indicates the best.

**Bleier dataset**	**Lab**	
**Method**	**Median**	**Mean**
Retinex (Funt et al., 2004)	2.68	3.40
Zhang (Zhang et al., 2016)	3.97	4.50
GIJ-GW (Arjan et al., 2012)	4.71	4.93
GIJ-GE1 (Arjan et al., 2012)	14.89	14.52
GU-GE1 (Gu et al., 2014)	3.39	3.32
GU-GW (Gu et al., 2014)	1.18	1.16
FG (Mutimbu and Robles-Kelly, 2016)	2.90	2.95
CC-CNN (Bianco et al., 2015)	3.32	3.51
DS-Net (Shi et al., 2016)	3.10	3.46
CN-DMS4	2.54	2.61

From Tables 1, 2, it can be seen that the mean error of the proposed method is reduced by 6.3% on the Gijsenij dataset and 15.5% on the MIMO dataset compared to the second best way.

It can be seen From Figure 4, from the first row of images, we find that the approximate shadow boundary can be accurately distinguished at the position of the illumination shadow boundary. Better fineness can be achieved in these scenes because our method is a step-by-step process; thus, we can accurately estimate the illumination position. In addition, there are a large number of synthetic images in the training datasets. The illumination boundary position of the synthetic color biased image is very similar to the light and shadows. Therefore, our method can deal with this boundary well. The images in the second column have more illumination colors, and almost every pixel given by the dataset is different. There is no such fine data in the training data, hence the estimated illumination is only consistent in the overall color. In addition, it is observed that the real illumination color in the training datasets is close to the color of the actual object surface in many areas and, in our method, it is difficult to accurately distinguish whether the color is that of the real illumination or the color of the object surface itself. However, it should be noted that the best existing MCC method must use gray-world to estimate the color of the light source. Gray-world is prone to different degrees of color deviation because of the color of the scene object itself. Because the high-precision dataset of multi-illumination scenes is limited, a learning-based method cannot learn the features well. Therefore, it can be considered that all known MCC-based methods have such problems, which may lead to color deviation. Further research is required to solve this problem with a small number of samples.

**Figure 5:**
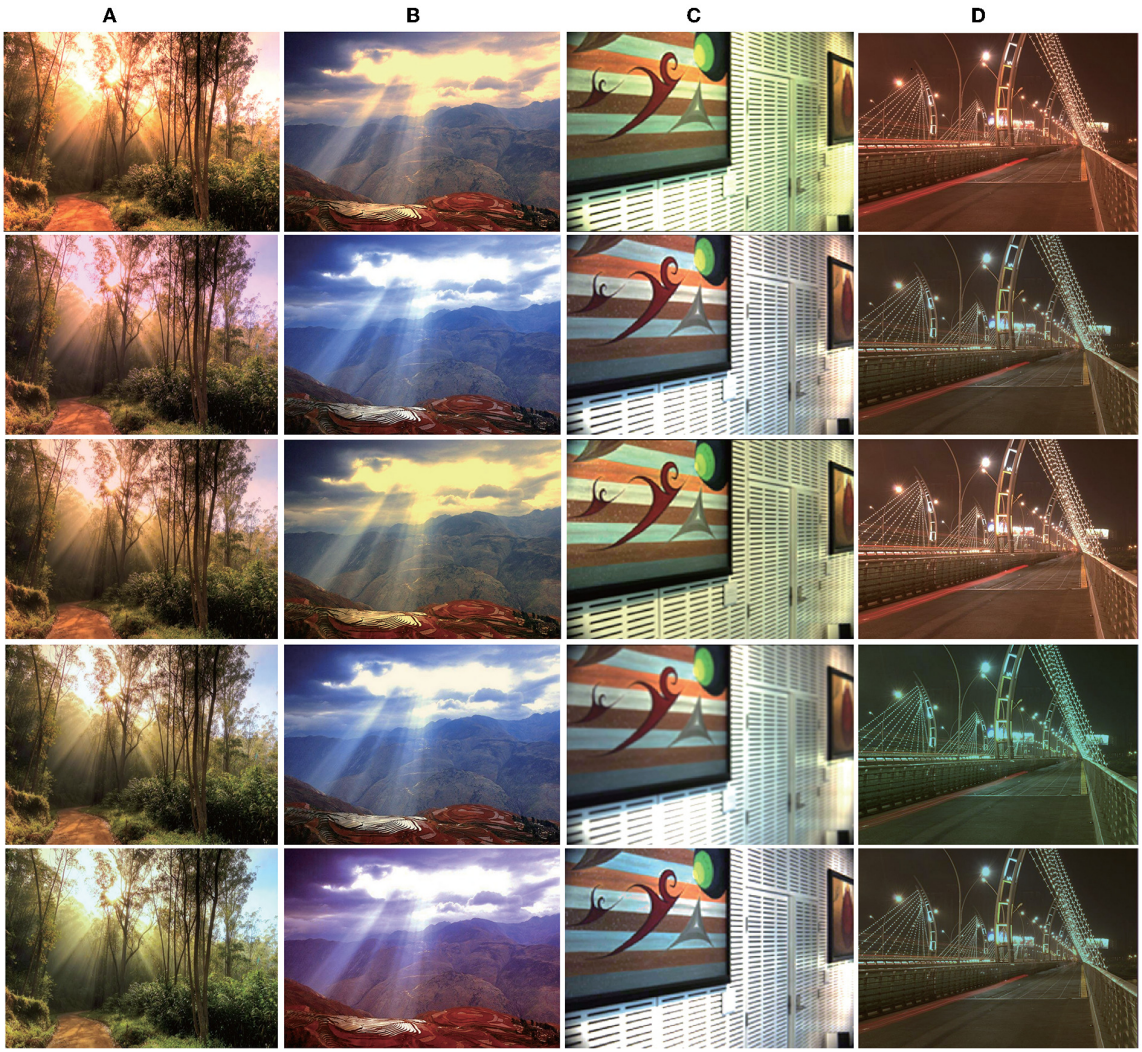
Qualitative results on natural scenes. The images in columns 1, 2, and 4 are taken from Baidu.com, available at: http://mms2.baidu.com/it/u=3199546478,84333290&fm=253&app=138&f=PNG&fmt=auto&q=75?w=669&h=500, http://mms0.baidu.com/it/u=576667012,1565892735&fm=253&app=138&f=JPEG&fmt=auto&q=75?w=500&h=331 and http://mms2.baidu.com/it/u=3592193920,2788102915&fm=253&app=138&f=JPEG&fmt=auto&q=75?w=500&h=329. The image in the third column is from the doctoral thesis (Gao, 2017). For each column, from top to bottom: Original image; **(A)** Result By GP (Yang et al., 2015); **(B)** Result By Retinex (Brainard and Wandell, 1986); **(C)** Result By Zhang (Zhang et al., 2016); **(D)** Our method.

In addition, we searched and downloaded several visual deviation images with multiple lighting from the Internet^3^. These color-biased images are corrected by different MCC methods. Because real illumination cannot be obtained, the effect of the corrected images can only be judged subjectively. Some correction comparison results are shown in Figure 5. As can be seen from the first row in the figure, these scenes contain a variety of lighting. Visually, the color deviation caused by a different illumination has been partially improved; for example, in the images in the first column, the light of the morning glow is yellow, which blocks the green of some trees. After our method, the trees and the sky are more real in visual effect. It can be seen from the images in the second and fourth columns that although other methods also eliminate part of the light, the overall color tone still shows color deviation visually. On applying our method, although the image still looks a little color biased, the image is more natural.

**Figure 6:**
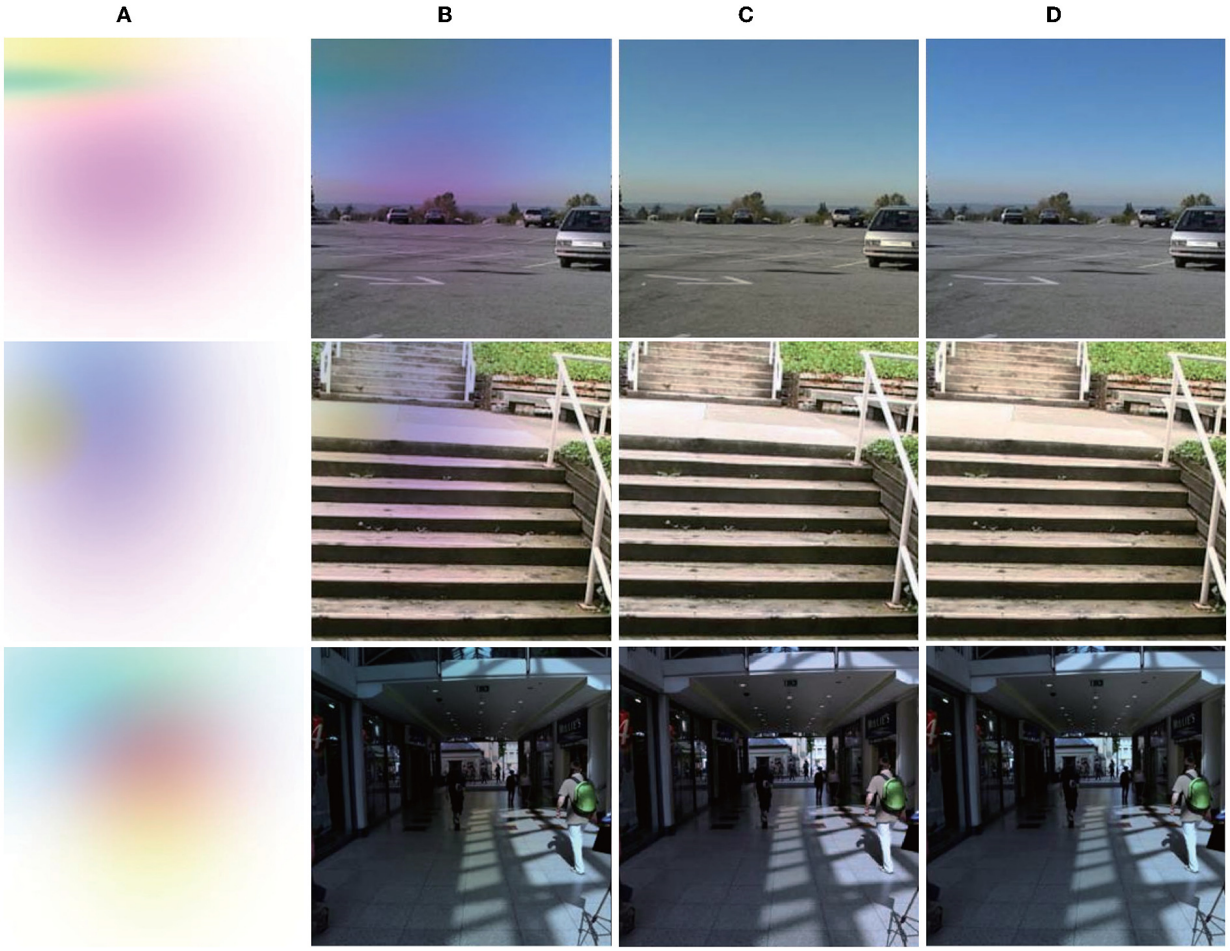
Results produced by the proposed approach on removing of multi-illuminant color cast, the images come from SFU Gray-Ball (Ciurea and Funt, 2003). From left to right: **(A)** Tint maps; **(B)** Synthesized images; **(C)** Predictions; **(D)** Ground truth. Dataset available at: https://www2.cs.sfu.ca/~colour/data/gray_ball/index.html. Reproduced with permission from Ciurea and Funt (2003).

As the lack of multi-illumination datasets, as an extension, we evaluate the proposed method using a tinted Multi-illuminant dataset (Sidorov, 2019) which is synthesized from the SFU Gray-Ball (Ciurea and Funt, 2003), this method not only synthesizes multiple lights but also synthesizes the superposition of multiple lights. Performance is quantitatively compared to the performance of state-of-the-art methods and is reported in Table 4, and some images are demonstrated for visual evaluation in Figure 6. It may be seen that the proposed technique outperforms all existing multi-illuminant algorithms. We observed that some images had slightly increased or reduced brightness, although the color cast is removed correctly.

**Table 4:** Quantitative evaluation on the tinted multi-illuminant dataset (Sidorov, 2019), red indicates the best.

**Method**	**Median**	**Mean**
GIJ-GW (Arjan et al., 2012)	6.61	10.50
GIJ-GE1 (Arjan et al., 2012)	6.70	12.10
GU-GE1 (Gu et al., 2014)	8.14	15.56
GU-GW (Gu et al., 2014)	5.51	9.78
CC-CNN (Bianco et al., 2015)	5.64	5.88
DS-Net (Shi et al., 2016)	6.19	7.66
FC4 (Hu et al., 2017)	4.27	4.89
CN-DMS4	3.42	3.71

### 3.5. Adaptation for single-illumination

**Figure 7:**
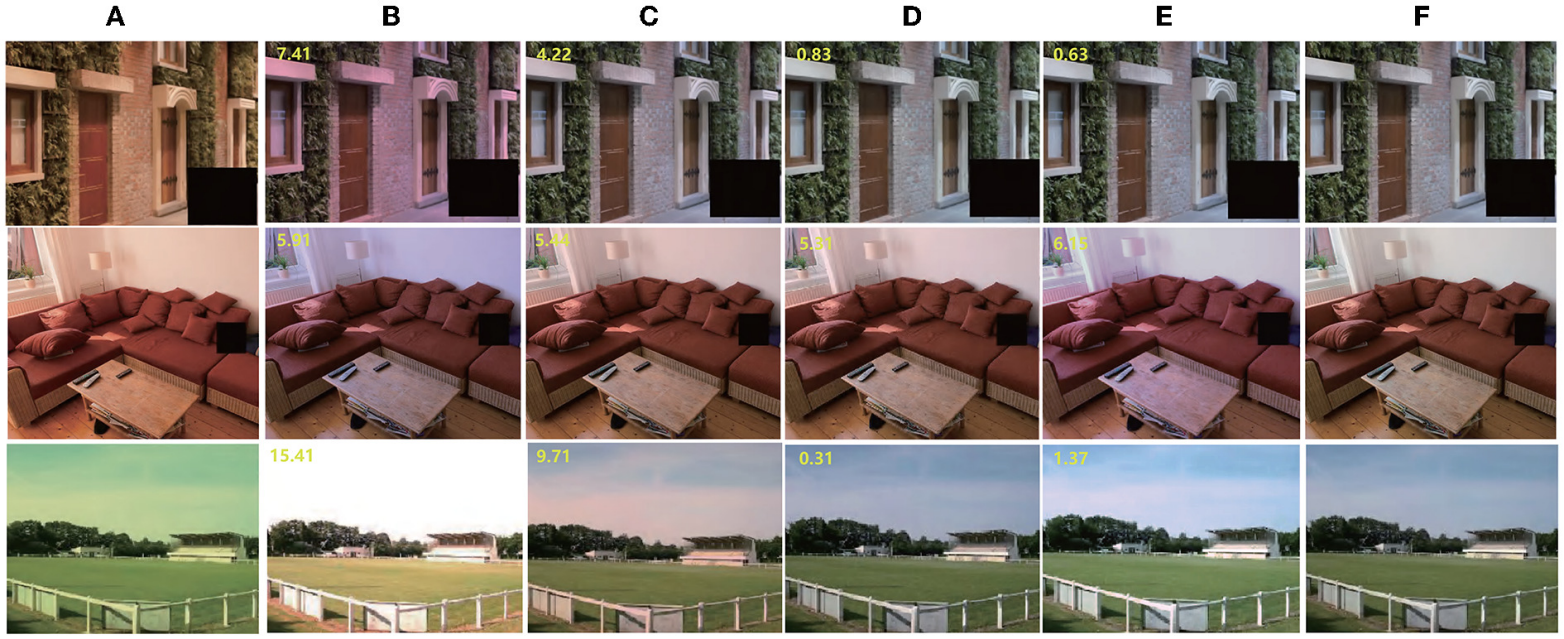
Result of single illumination, the images come from ADE20k (Zhou et al., 2016). From left to right: **(A)** Original image; **(B)** Result by DS-Net (Shi et al., 2016); **(C)** Result by FC4 (Hu et al., 2017); **(D)** Result by MSRWNS (Wang et al., 2022); **(E)** Result by proposed method; **(F)** Ground truth. Dataset available at: https://groups.csail.mit.edu/vision/datasets/ADE20K/. Reproduced with permission from Zhou et al. (2016).

As mentioned in this paper, the proposed method aims to solve the color constancy problem under multiple illuminations, and we mainly compare it with existing MCC methods and with methods that can estimate local illumination. For single-illumination, we added some single-illumination datasets and used the same illumination as illumination maps for training. we take the mean value of the illumination map as the estimated illumination, and compare it with three single-illumination methods: DS-Net (Shi et al., 2016), FC4 (Hu et al., 2017), and our previous single-illumination method, MSRWNS (Wang et al., 2022). The quantitative performance comparison of the SFU Gray-Ball dataset (Ciurea and Funt, 2003) and ADE20k dataset (Zhou et al., 2016) are presented in Tables 5, 6, some results are shown in Figure 7. It may be seen that the proposed method also shows a better performance in single-light estimation, second only to our previous method.

**Table 5:** Quantitative evaluation on SFU Gray-Ball (Ciurea and Funt, 2003), red indicates the best.

**Method**	**Median**	**Mean**
DS-Net (Shi et al., 2016)	0.96	2.41
FC4 (Hu et al., 2017)	1.12	2.33
MSRWNS (Wang et al., 2022)	0.82	1.83
CN-DMS4	0.95	2.24

**Table 6:** Quantitative evaluation on ADE20k (Zhou et al., 2016), red indicates the best.

**Method**	**Median**	**Mean**
DS-Net (Shi et al., 2016)	0.96	1.68
FC4 (Hu et al., 2017)	1.32	1.56
MSRWNS (Wang et al., 2022)	0.61	1.68
CN-DMS4	1.13	0.95

### 3.6. Efficiency

The code used to test the efficiency of the proposed method is based on PyTorch (Paszke et al., 2019) and the training took approximately 8 h, after which the loss tended to stabilize. In the testing phase, we used OpenCV (Bradski, 2000) to load the model. An average image required 200 ms on a CPU, and 32 ms on a GPU ^4^. For low-resolution images, the real-time estimation can be achieved using a GPU, but for high-resolution images, the algorithm requires significant time. In the future study, we will try to prune the model to further improve its efficiency.

## 4. Conclusion

Most studies of color constancy are based on the assumption that there is only a single-illumination in the scene. However, in reality, most scenes have more than one illumination. For the illumination estimation in this study, the encoding and decoding network was introduced, and a unique network model of multiscale supervision and single-scale estimation was designed. An optimization network with an improved loss function and a simple operator with a penalty was designed to train the network. By testing on several public datasets, our method yielded a partial improvement in terms of quantitative data and visual effects compared with previous multi-illumination estimation methods. This provides a research direction in end-to-end multi-illumination estimation.

## Data availability statement

The original contributions presented in the study are included in the article/supplementary material, further inquiries can be directed to the corresponding author.

## Author contributions

FW is responsible for conceptualization, investigation, data curation, and writing. WW is responsible for formal analysis, investigation, and methodology. DW is responsible for formal analysis, investigation, and validation. GG is responsible for data curation and investigation. ZW is responsible for polishing the language and the major experiments in the revised version. All authors contributed to the article and approved the submitted version.

## Funding

The Project supported by the Science Fund of State Key Laboratory of Advanced Design and Manufacturing for Vehicle Body (No. 32015013) and the Shaanxi Province Key R&D Program Project (No. 2022GY-435).

## Conflict of interest

The authors declare that the research was conducted in the absence of any commercial or financial relationships that could be construed as a potential conflict of interest.

## Publisher's note

All claims expressed in this article are solely those of the authors and do not necessarily represent those of their affiliated organizations, or those of the publisher, the editors and the reviewers. Any product that may be evaluated in this article, or claim that may be made by its manufacturer, is not guaranteed or endorsed by the publisher.

## Footnotes

1 We assume that there are multiple light sources in the scene and that the illumination of the multiple light sources is uniform.

2 As demonstrated in the literature (Finlayson et al., 2004; Barron, 2015), log-uv has advantages over *RGB*. First, there are two variables instead of three. Second, the multiplicative constraint of the illumination estimation model is converted to a linear constraint.

3 https://image.baidu.com

4 Experimental hardware platform: Intel Xeon Silver 4210R, 64-GB memory, GTX3090. The resolution of the test image was less than 800*600.
